# An Algorithm for the Pricing and Timing of the Option to make a Two-Stage Investment with Credit Guarantees

**DOI:** 10.1007/s10614-021-10220-8

**Published:** 2021-11-27

**Authors:** Linjia Dong, Zhaojun Yang

**Affiliations:** 1 School of Mathematics, Harbin Institute of Technology, Harbin, China; 2 Department of Finance, Southern University of Science and Technology, Shenzhen, 518055 China

**Keywords:** Entrepreneurship, Guarantee-investment mode, Growth options, Jump-diffusion, Algorithms, G12, H81, G31

## Abstract

We develop a jump-diffusion model for a guarantee-investment combination financing mode (G-I mode) that is recently popular in financial practice. We assume that a borrower has exclusively an option to invest in a project in two stages. The project’s cash flow follows a double exponential jump-diffusion process and it is increased by a growth factor once the second-stage investment is exercised. The first-stage investment cost is financed by a bank loan with the guarantee provided by an insurer, who promises to provide the second-stage investment cost as well as take the lender’s all default losses. In return for the guarantee and investment, the borrower pays a guarantee fee upon first investment and grants a fraction of equity upon second investment to the insurer. In sharp contrast to prior papers on guarantee, the guarantee costs are contracted prior to investment. We provide closed-form solutions and produce a numerical algorithm for the timing and pricing of the two investment options.

## Introduction

A new financing mode called guarantee-investment mode (G-I mode, henceforth) is recently invented. The G-I mode appears because of two main factors as follows. On the one hand, to support micro-, small- and medium-sized enterprises (MSMEs), Chinese governments demand that insurers provide guarantees for MSMEs to get a loan from a bank and the guarantee fee must be less than 3% or so of the money borrowed. On the other hand, most of projects managed by MSMEs are exposed to very high risk, and the guarantee fee rate is too low to cover the default losses incurred by insurers. To solve this problem, Shenzhen High-Tech Investment Group Co., Ltd. (HTI henceforth) has invented the G-I mode.

As we know, many financial investments in a project are managed by trial and error and can be roughly divided into two stages: The investment is first exercised by trial on a small scale and if successful, a large-scale investment follows. For this reason, HTI develops the G-I financing mode. Under this mode, the first-stage investment cost is financed by a bank loan with the guarantee provided by an insurer, who promises to provide the second-stage investment cost as well as take the lender’s all default losses. In return for the guarantee and investment, the borrower pays the insurer a guarantee fee at the first investment time and a fraction of equity at the second investment time. Due to the fraction of equity granted to the insurer, which is valuable in general, the insurer will be sufficiently reimbursed while the guarantee fee rate satisfies the standard required by the government.

According to Dong and Yang (2020), HTI is the first group of local brands in China and has made fruitful achievements with the G-I mode. Thanks to the G-I mode, 10 enterprises have been listed in the A-share market, 3 listed in the H-share market, and 5 listed in M&A. HTI supported MSMEs such as Han’s Laser Technology Industry Group Co., Ltd., Ofilm Group Co., Ltd., Skyworth Group Co., Ltd., Dongjiang Environment Co., Ltd., and Sinofibers Technology Co., Ltd. to develop into industry leaders.

This paper considers the pricing and timing of the option to invest in a project in two stages. The sunk costs are financed with the guarantee-investment mode. We assume that the cash flow of the project follows a double exponential jump-diffusion process and will be increased by a given growth factor immediately after the second-stage investment has taken place. The first-stage investment cost is financed by a bank loan with the guarantee provided by an insurer, who promises to provide the second-stage investment cost as well as take the lender’s all default losses. In return for the guarantee and investment, the borrower pays the insurer a guarantee fee in cash when the first-stage investment cost is financed, as well as grants the insurer a fraction of equity when the second-stage investment is conducted.

We emphasize that in sharp contrast to prior papers on credit guarantees, the guarantee agreement is signed prior to investment. That is, similar to the loan commitment discussed by Mauer and Sarkar (2005) and Song and Yang (2016), borrowers, lenders, and insurers enter into the guarantee agreement in advance which specifies the guarantee fee rate and the fraction of equity granted to insurers. Particularly, two investment thresholds are not contracted and they are totally determined by borrowers. The loan is risk-free and thus this is essentially a game between borrowers and insurers and the fair guarantee is a Nash equilibrium of the game.

To highlight the pricing and timing of the two-stage investment option, we assume that the guarantee market is fully competitive and thus the net present value of the insurer is zero. After complicated computations, we successfully provide closed-form solutions and produce a numerical algorithm for the timing and pricing of the real option. To explain that our algorithms are effective, we provide numerical examples.

*Literature review* This work aims to solve a complicated computational problem arising from the pricing and timing of the real option to invest in a project, in which the investment is divided into two stages. There is a long research line on the real options theory that originates from the work of Myers (1977) and is becoming more and more popular. Considerable contributions addressing the theoretical aspect of real options are made by McDonald and Siegel (1986), Dixit et al. (1994) and others.

This paper considers an entrepreneur having a real option, of which the sunk cost is financed with loan guarantees. The real options are related to the interaction between investment and financing decisions. The research on loan guarantees originates with Merton (1977) and recent work is due to Bachas et al. (2021) among others. However, the guarantees of this line are bought by lenders instead of borrowers as done in a lot of financial practice and assumed by our model. In contrast to ours, their guarantee contracts are more similar to credit default swaps (CDSs).

Credit guarantees develop gradually and have a long history. Credit guarantee system dates back to the 1930s when the global economic crisis happened. However, to the best of our knowledge, the theoretical models on loan guarantees bought by borrowers develop relatively late and date back to Yang and Zhang (2013) addressing a special guarantee mode called equity-for-guarantee swaps. After that, credit guarantees under different situations have been widely considered by Yang and Zhang (2013), Xiang and Yang (2015), Yang and Zhang (2015a, b), Wang et al. (2015), Luo et al. (2016), Gan et al. (2016), Tang and Yang (2017, 2018), Luo and Yang (2019) and Yang (2020).

The equity-for-guarantee swaps are related to CDSs, which have been widely studied in the literature, but they have major differences. The equity-for-guarantee swaps are mainly for MSMEs who must get a guarantee *in advance* to obtain bank loans. The equity-for-guarantee swaps are impossible to be replaced by credit default swaps (CDSs) since the latter are signed between insurers and *lenders instead of borrowers*. In particular, the buyers of CDS insurance are generally large corporations rather than MSMEs in the equity-for-guarantee swaps. With CDSs, lenders pay regular premiums for hedging risk until a credit event. By contrast, equity-for-guarantee swaps allow lenders to transfer risk to insurers at the cost of an MSME’s one and only one lump-sum payment in the form of capital or equity, which motivates the lenders to grant loans to MSMEs. Hence, the equity-for-guarantee swaps give rise to a large welfare improvement to the economy. All in all, without such equity-for-guarantee swaps, MSMEs might be unable to start a profitable project. By sharp contrast, CDSs mainly involve shifting of risk.

This paper is most closely related to Dong and Yang (2020) since the two papers consider the same guarantee mode, i.e. the G-I mode. The major differences are as follows. We assume that the cash flow of the project follows a double exponential jump-diffusion distribution rather than an arithmetic Brownian motion discussed by the latter. This difference makes our model much more difficult to solve. For example, one main task is to define fair guarantee costs which depend on the cash flow level when the agreement is signed. However, the cash flow level at investment time is uncertain due to jumps assumed in this paper but it can be an optimally chosen level in advance in Dong and Yang (2020) since jumps are absent there. Actually, there is a new challenging problem that is never considered in prior papers on the guarantee. For this reason, this paper mainly aims to provide an algorithm to fix the pricing and timing of the option to invest in a jump-diffusion model with the G-I mode.

The jump-diffusion model is widely used in the financial literature. This is because many empirical studies have shown that financial returns exhibit significantly fatter tails than standard normal models. To effectively improve the latter, roughly speaking, there are two approaches: One is to develop stochastic volatility models and the other is to introduce stochastic jumps, setting up jump-diffusion models, say one introduced by Merton (1976). As a matter of fact, stochastic jumps occur more often than not in a period. For example, in the COVID-19 pandemic, many MSMEs have experienced financial distress and thus their cash flows experience a sharp decline. Recently, the double exponential jump-diffusion process, a new jump-diffusion model, attracts a growing research interest due to its two merits, as argued by (Kou 2002): First, its two-sided jumps and the leptokurtic feature of jump size lead to the peak and heavy tails of return distribution found in reality. Second, the double exponential distribution has a memoryless feature which facilitates the calculation of conditional means and variances. For these reasons, in this paper we focus on the double exponential jump-diffusion process instead of the pure diffusion model discussed by Dong and Yang (2020).

The remainder of the paper is organized as follows. Section 2 sets up the model. Section 3 addresses the pricing and timing of the option to make the second-stage investment. Section 4 discusses the pricing and timing of the option to make the first-stage investment, the forward-like claim, and the fair guarantee-investment combination agreement. Section 5 provides the algorithm and examples. Section 6 concludes.

## Model Setup

Following Dong and Yang (2020), we consider an entrepreneur who has an option to make an irreversible but delayable investment with the initial sunk cost *I*. In addition, the entrepreneur has an American growth investment option with the sunk cost ${\bar{I}}$. The cash flow of the investment is observable and does not depend on the firm’s capital structure. In contrast to Dong and Yang (2020), following the common continuous-time model as studied in Yang and Zhang (2015b), we assume the cash flow $\delta$ after the first-stage investment but prior to the second-stage investment follows the double exponential jump diffusion process, i.e

1 $$\begin{aligned} d\delta _{t}=\mu dt+\sigma dW_t+d\left( \sum _{i=1}^{N_{t}}Z_i\right) ,\quad t\in \left[ \tau _0,\infty \right) , \end{aligned}$$

where $\mu$ is a constant risk-adjusted growth rate, $\sigma$ is a constant volatility, and the process *W* is a standard Brownian motion. For the jump part, *N* is a Poisson process with a constant intensity rate $\lambda > 0$, and $\left\{ Z_{i},\ i=1,2,...\right\}$ denote independent and identically distributed random variables following a double exponential distribution, of which the density function is given by

$$\begin{aligned} h(z)=p\cdot \eta _{1}e^{-\eta _{1}z}{} \mathbf{{1}}_{\left\{ z\ge 0\right\} }+q\cdot \eta _{2}e^{\eta _{2}z}{} \mathbf{{1}}_{\left\{ z< 0\right\} }, \end{aligned}$$

where *p*, $q\ge 0$ representing the probabilities of upward and downward jumps, are constants, $p+q=1$, and $\eta _{1},\eta _{2}>0$. Noting that the means of the two exponential distributions are $\frac{1}{\eta _{1}}$ and $\frac{1}{\eta _{2}}$ respectively, we therefore have $\xi \equiv {\mathbb {E}}(Z_{i})=\frac{p}{\eta _{1}}-\frac{q}{\eta _{2}}$.

Following Dong and Yang (2020), an entrepreneur (or MSME) has two investment options: one is the first-stage investment and the other is the second-stage investment. The MSME must borrow money from an lender (bank) under the guarantee provided by an insurer for the first-stage investment and pay the bank a coupon rate of *C* unless it is solvent. If the borrower defaults, the insurer takes the firm’s liquidation value with the bankruptcy loss rate being denoted by $0<\alpha <1$, and compensates the lender for all default loss incurred. In return, the MSME pays the insurer a given guarantee fee at the time the loan is issued and also gives the insurer a forward-like claim, which claims that if the second-stage investment is conducted, the entrepreneur grants the insurer a given fraction $\psi$ of equity after the insurer pays the sunk cost ${\bar{I}}$ of the second-stage investment. As assumed by Dong and Yang (2020), once the second-stage investment is exercised, the MSME’s cash flow level increases from $\delta$ to $(1+\theta )\delta$, where $\theta$ represents the growth factor coefficient. If the cash flow level is low enough, the MSME will default no matter whether the second-stage investment is executed or not.

We note that our model is time-homogeneous. Therefore, all the decisions are independent of time and all the optimal investment thresholds and default thresholds must be independent of time though they would depend on the cash flow level when the first-stage investment is exercised. For this reason, the initial and growth investment thresholds are denoted by $\delta ^I$ and $\delta ^{{\bar{I}}}$ respectively; the default thresholds before and after the second-stage investment are denoted by $\delta ^{B}$ and $\delta ^{{\bar{B}}}$ respectively. In addition, we assume the current time is zero in the following text. This assumption does not lose generality.

Following Dong and Yang (2020) among many others, we adopts a simple tax rate structure. The interest tax rate is denoted by $\rho _i$, the dividend tax rate is $\rho _d$, and the corporate profit tax rate is $\rho _c$. Let $1 - \rho _e = (1 - \rho _d) (1 - \rho _c)$, where $\rho _e$ represents the corporate effective tax rate.

## The Pricing and Timing of the Option to make the Second-Stage Investment

To fix the pricing and timing of the option to invest in a project in two stages, we follow Yang and Zhang (2015b) and take a backward induction approach. We first consider the growth (second-stage) investment and then turn to the first-stage investment. Doing so, we first assume that a fair guarantee contract, investment thresholds, default thresholds, and the cash flow level when the first-stage investment is exercised are given and then fix the fair guarantee agreement and these thresholds by solving some proper optimization problems.

### The Pricing of Contingent Claims after the Second-Stage Investment

Clearly, there are two firm securities after the second-stage investment: one is the debt with the coupon rate being constant *C* and the other is equity with cash flow being $(1+\theta )x-C$ if the current cash flow level $x \in {\mathcal {D}} = \left( \delta ^{{\bar{B}}},+\infty \right)$, i.e. the firm is solvent.

We denote by $E_2(x)$ and $D_2(x)$ the value of equity and debt respectively. Naturally, they are the function of the current cash flow level *x*.

For easy of exposition, we introduce the Laplace exponent $H(\cdot )$ of cash flow $\delta$ such that ${\mathbb {E}} \left[ e^{\beta \delta _t}\right] = \exp \left[ H(\beta )t\right]$. Then we have

$$\begin{aligned} H(\beta )=\frac{\sigma ^2\beta ^2}{2}+\mu \beta -\frac{\lambda p\eta _{1}}{\beta -\eta _{1}}+\frac{\lambda q \eta _{2}}{\beta +\eta _{2}}-\lambda . \end{aligned}$$

The equation $H(\beta ) = r$ has four roots: $\beta _{1}$, $\beta _{2}$, $-\beta _{3}$, $-\beta _{4}$, satisfying $-\infty< -\beta _{4}< -\eta _{2}< -\beta _{3}< 0< \beta _{1}< \eta _{1}< \beta _{2} < \infty$. For more details, please refer to Kou and Wang (2003).

In addition, define $a_g\equiv \frac{(1-\rho _e)(1-\alpha )(1+\theta )}{r}$, $b_g\equiv \frac{(1-\rho _e)(1-\alpha )(1+\theta )(\mu +\lambda \xi )}{r^2}$ and $\delta ^{{\bar{B}}}_0\equiv -\frac{\mu +\lambda \xi }{r}$. Thanks to these denotations, we have the following conclusions. We emphasize that once the current cash flow is less than $\delta ^{{\bar{B}}}_0$, the firm should default shortly and what’s more, the liquidation value is negative. Therefore, both the value of equity and that of debt are assumed to be zero due to the limited liability.

#### Proposition 3.1

After the second-stage investment has taken place, for a given default threshold $\delta ^{{\bar{B}}}$, if the firm is solvent, then the value $E_2(x)$ of equity is given by

2 $$\begin{aligned} E_2(x)= A_3^{*}e^{-\beta _{3}(x-\delta ^{{\bar{B}}})}+A_4^{*}e^{-\beta _{4}(x-\delta ^{{\bar{B}}})}+E_2^{0}, \end{aligned}$$

where

3 $$\begin{aligned} \left\{ \begin{array}{l} A_3^{*}=\frac{(1+\theta )(1-\rho _e)(\eta _{2}-\beta _{3})}{r\eta _{2}(\beta _{3}-\beta _{4})}\left( 1-\frac{C}{1+\theta }\beta _{4}+\beta _{4}\delta ^{{\bar{B}}}-\frac{\beta _{4}}{\eta _{2}}+\frac{(\mu +\lambda \xi )\beta _{4}}{r}\right) ,\\ A_4^{*}=\frac{(1+\theta )(1-\rho _e)(\eta _2-\beta _4)}{r\eta _2(\beta _4-\beta _3)}\left( 1-\frac{C}{1+\theta }\beta _3+\beta _3\delta ^{{\bar{B}}}-\frac{\beta _3}{\eta _2}+\frac{(\mu +\lambda \xi )\beta _3}{r}\right) ,\\ E_2^{0}\equiv (1-\rho _e)\left( \frac{(1+\theta )x}{r}-\frac{C}{r}+(1+\theta )\frac{\mu +\lambda \xi }{r^2}\right) . \end{array}\right. \end{aligned}$$

The value $D_2(x)$ of debt is given by

4 $$\begin{aligned} D_2(x)= B_3^{*}e^{-\beta _{3}(x-\delta ^{{\bar{B}}})}+B_4^{*}e^{-\beta _{4}(x-\delta ^{{\bar{B}}})}+D_2^{0}, \end{aligned}$$

where

5 $$\begin{aligned} \left\{ \begin{array}{l} B_3^{*}=\frac{\eta _{2}-\beta _{3}}{(\beta _{4}-\beta _{3})\eta _{2}}\left[ \left( a_g\delta ^{{\bar{B}}}+b_g-D_2^0 \right) \beta _{4}+\frac{a_g(\eta _{2}-\beta _{4})}{\eta _{2}}(1-e^{-\eta _{2}(\delta ^{{\bar{B}}}-\delta ^{{\bar{B}}}_0)})\right] ,\\ B_4^{*}=\frac{\eta _{2}-\beta _{4}}{(\beta _{3}-\beta _{4})\eta _{2}}\left[ \left( a_g\delta ^{{\bar{B}}}+b_g-D_2^0 \right) \beta _{3}+\frac{a_g(\eta _{2}-\beta _{3})}{\eta _{2}}(1-e^{-\eta _{2}(\delta ^{{\bar{B}}}-\delta ^{{\bar{B}}}_0)})\right] ,\\ D_2^{0}\equiv \frac{(1-\rho _i)C}{r}. \end{array} \right. \end{aligned}$$

The optimal default-triggering level $\delta ^{{\bar{B}}}$ chosen by the entrepreneur is given by

6 $$\begin{aligned} \delta ^{{\bar{B}}}=\frac{C}{1+\theta }-\frac{\mu +\lambda \xi }{r}-\frac{1}{\beta _{3}}-\frac{1}{\beta _{4}}+\frac{1}{\eta _{2}}. \end{aligned}$$

#### Proof

To determine the value of an asset, we should first specify the cash flow generated by the asset. The cash flow of equity is $(1-\rho _e)\left[ (1+\theta )x - C\right]$ if the firm’s cash flow $x \in {\mathcal {D}} = \left( \delta ^{{\bar{B}}},+\infty \right)$ and it is zero forever once the firm defaults, i.e. the cash flow is less than $\delta ^{{\bar{B}}}$. Consequently, if the firm is solvent, i.e. the current cash flow level $x>\delta ^{{\bar{B}}}$, the value of equity is given by

$$\begin{aligned} E_2(x) = {\mathbb {E}}\left[ \int _{0}^{\tau _{{\mathcal {D}}}} e^{-rs}(1-\rho _e)((1+\theta )\delta _s - C) ds +e^{-r\tau _{{\mathcal {D}}}}G(\delta _{\tau _{{\mathcal {D}}}})\,\mid \,\delta _0=x\right] , \end{aligned}$$

where $\tau _{{\mathcal {D}}}$ is the first time for the cash flow to depart from the domain ${\mathcal {D}}$ and function $G(\cdot )$ is defined by $G(x)=0$, if $x\le \delta ^{{\bar{B}}}$. Using a method similar to Bellman’s principle of optimality, we obtain the value $E_2(x)$ of equity satisfying

$$\begin{aligned} rE_2(x)= & {} \frac{\mu \partial E_2(x)}{\partial x}+\frac{\sigma ^2\partial ^2 E_2(x)}{2\partial x^2}+(1-\rho _e)[(1+\theta )x - C]\\&+\lambda \int _{-\infty }^{+\infty }\left[ E_2(x+z)-E_2(x)\right] h(z)dz, \end{aligned}$$

that is,

7 $$\begin{aligned}&\mu \frac{\partial E_2(x)}{\partial x}+\frac{\sigma ^2}{2}\times \frac{\partial ^2 E_2(x)}{\partial x^2}+(1-\rho _e)[(1+\theta )x - C]\nonumber \\&\quad =(r+\lambda )E_2(x)-\lambda \int _{\delta ^{{\bar{B}}}-x}^{+\infty } E_2(x+z) h(z)dz \end{aligned}$$

According to corporate security pricing theory, we guess that the solution of (7) has the form of (2), where $A_3^*$ and $A_4^*$ are constants to be determined. Then we have

8 $$\begin{aligned}&\frac{\partial E_2(x)}{\partial x}=-A_3^*\beta _{3}e^{-\beta _{3}(x-\delta ^{{\bar{B}}})}-A_4^*\beta _{4}e^{-\beta _{4}(x-\delta ^{{\bar{B}}})}+\frac{(1-\rho _e)(1+\theta )}{r}, \end{aligned}$$

9 $$\begin{aligned}&\frac{\partial ^2 E_2(x)}{\partial x^2}=A_3\beta _{3}^2e^{-\beta _{3}(x-\delta ^{{\bar{B}}})}+A_4\beta _{4}^2e^{-\beta _{4}(x-\delta ^{{\bar{B}}})}, \end{aligned}$$

and

10 $$\begin{aligned} \int _{\delta ^{{\bar{B}}}-x}^{+\infty } E_2(x+z) h(z)dz=\int _{\delta ^{{\bar{B}}}-x}^{0} E_2(x+z) q\cdot \eta _{2}e^{\eta _{2}z}dz+\int _{0}^{+\infty } E_2(x+z) p\cdot \eta _{1}e^{-\eta _{1}z}dz, \end{aligned}$$

where

$$\begin{aligned}&\int _{\delta ^{{\bar{B}}}-x}^{0} E_2(x+z) q\cdot \eta _{2}e^{\eta _{2}z}dz\\&\quad =\int _{\delta ^{{\bar{B}}}-x}^{0}\left[ q\cdot \eta _{2}A_3^*e^{-\beta _{3}(x-\delta ^{{\bar{B}}})+(\eta _{2}-\beta _{3})z}+q\cdot \eta _{2}A_4^*e^{-\beta _{4}(x-\delta ^{{\bar{B}}})+(\eta _{2}-\beta _{4})z}\right] dz\\&\qquad +\int _{\delta ^{{\bar{B}}}-x}^{0}\left[ (1-\rho _e)\left( \frac{(1+\theta )(x+z)}{r}-\frac{C}{r}+(1+\theta )\frac{\mu +\lambda \xi }{r^2}\right) q\cdot \eta _{2}e^{\eta _{2}z}\right] dz\\&\quad =- \frac{q\eta _{2}A_3^*}{\beta _{3}-\eta _{2}}\left( e^{-\beta _{3}(x-\delta ^{{\bar{B}}})}-e^{-\eta _{2}(x-\delta ^{{\bar{B}}})}\right) -\frac{q\eta _{2}A_4^*}{\beta _{4}-\eta _{2}}\left( e^{-\beta _{4}(x-\delta ^{{\bar{B}}})}-e^{-\eta _{2}(x-\delta ^{{\bar{B}}})}\right) \\&\qquad +\frac{q(1-\rho _e)(1+\theta )}{r}\left[ \left( \frac{1}{\eta _{2}}-\frac{\mu +\lambda \xi }{r}+\frac{C}{(1+\theta )} \right) \right. \\&\qquad \left. (e^{\eta _{2}(\delta ^{{\bar{B}}}-x)}-1)+x-\delta ^{{\bar{B}}}e^{\eta _{2}(\delta ^{{\bar{B}}}-x)} \right] , \end{aligned}$$

and

$$\begin{aligned}&\int _{0}^{+\infty } E_2(x+z) p\cdot \eta _{1}e^{-\eta _{1}z}dz\\&\quad =\frac{p\eta _{1}A_3^*}{\beta _{3}+\eta _{1}} e^{-\beta _{3}(x-\delta ^{{\bar{B}}})}+\frac{p\eta _{1}A_4^*}{\beta _{4}+\eta _{1}} e^{-\beta _{4}(x-\delta ^{{\bar{B}}})}\\&\qquad +\frac{p(1-\rho _e)(1+\theta )}{r}\left[ x-\frac{C}{(1+\theta )}+\frac{\mu +\lambda \xi }{r}+\frac{1}{\eta _{1}}\right] . \end{aligned}$$

Substituting (8), (9) and (10) into (7), we get that

11 $$\begin{aligned} \chi _1e^{-\beta _{3}(x-\delta ^{{\bar{B}}})}+\chi _2e^{-\beta _{4} (x-\delta ^{{\bar{B}}})}+\chi _3e^{-\eta _{2}(x-\delta ^{{\bar{B}}})}=0 \end{aligned}$$

holds for all $x>\delta ^{{\bar{B}}}$, where

$$\begin{aligned} \left\{ \begin{array}{l} \chi _1=-\mu A_3^*\beta _{3}+\frac{1}{2}\sigma ^2A_3^*\beta _{3}^2+\frac{\lambda p\eta _{1}A_3^*}{\beta _{3}+\eta _{1}}-\frac{\lambda q \eta _{2}A_3^*}{\beta _{3}-\eta _{2}}-\lambda A_3^*-rA_3^*=A_3^*[H(-\beta _3)-r]=0,\\ \chi _2=-\mu A_4^*\beta _{4}+\frac{1}{2}\sigma ^2A_4^*\beta _{4}^2+\frac{\lambda p\eta _{1}A_4^*}{\beta _{4}+\eta _{1}}-\frac{\lambda q \eta _{2}A_4^*}{\beta _{4}-\eta _{2}}-\lambda A_4^*-rA_4^*=A_4^*[H(-\beta _4)-r]=0,\\ \chi _3=q\lambda \left[ \frac{\eta _{2}A_3^*}{\beta _{3}-\eta _{2}}+\frac{\eta _{2}A_4^*}{\beta _{4}-\eta _{2}}+\frac{(1-\rho _e)(1+\theta )}{r} \left( \frac{1}{\eta _{2}}-\frac{\mu +\lambda \xi }{r}+\frac{C}{(1+\theta )} -\delta ^{{\bar{B}}}\right) \right] . \end{array} \right. \end{aligned}$$

Therefore, we have $\chi _3=0$ also due to (11). In addition, thanks to the value-matching condition, we conclude from (2) that constants $A_3^*$ and $A_4^*$ are the solution of the following system of linear equations:

$$\begin{aligned} {\left\{ \begin{array}{ll} \frac{\eta _{2}A_3^*}{\beta _{3}-\eta _{2}}+\frac{\eta _{2}A_4^*}{\beta _{4}-\eta _{2}}+\frac{(1-\rho _e)(1+\theta )}{r} \left( \frac{1}{\eta _{2}}-\frac{\mu +\lambda \xi }{r}+\frac{C}{(1+\theta )} -\delta ^{{\bar{B}}}\right) =0,\\ A_3^{*}+A_4^{*}+(1-\rho _e)\left( \frac{(1+\theta )\delta ^{{\bar{B}}}}{r}-\frac{C}{r}+(1+\theta )\frac{\mu +\lambda \xi }{r^2}\right) =0. \end{array}\right. } \end{aligned}$$

Solving it leads to (3).

Now we turn to the pricing of debt. Clearly, at a given coupon payment *C*, the value of debt is determined entirely by default threshold $\delta ^{{\bar{B}}}$.

Hence, the value $D_2(\cdot )$ of debt can be expressed as follows:

$$\begin{aligned} D_2(x)={\mathbb {E}} \left[ \int _{0}^{\tau _{{\mathcal {D}}}} e^{-rs} (1-\rho _i)C \,ds +e^{-r\tau _{{\mathcal {D}}}}G(\delta _{\tau _{{\mathcal {D}}}})\,\mid \,\delta _0=x\right] ,\ x\ge \delta ^{{\bar{B}}}, \end{aligned}$$

where function $G(\cdot )$ is defined by $G(x)=\left[ (1-\rho _e)(1-\alpha )(1+\theta )\frac{\mu +\lambda \xi +rx}{r^2}\right] \vee 0$,^1^ for $x\le \delta ^{{\bar{B}}}$.

Thanks to It${\hat{o}}$’s formula, we derive that debt value $D_2(x)$ satisfies the equation:

12 $$\begin{aligned} \mu \frac{\partial D_2(x)}{\partial x}+\frac{\sigma ^{2}}{2}\times \frac{\partial ^2 D_2(x)}{\partial x^2}+(1-\rho _i)C+\lambda \int _{\delta ^{{\bar{B}}}-x}^{+\infty } D_2(x+z) h(z)dz=(\lambda +r)D_2(x). \end{aligned}$$

In the same way, we guess that the solution of (12) has the form:

13 $$\begin{aligned} D_2(x)= {\left\{ \begin{array}{ll} 0, &{} x \le \delta ^{{\bar{B}}}_0,\\ (1-\rho _e)(1-\alpha )(1+\theta )\frac{\mu +\lambda \xi +rx}{r^2}, &{} \delta ^{{\bar{B}}}_0<x\le \delta ^{{\bar{B}}},\\ B_3^{*}e^{-\beta _{3}(x-\delta ^{{\bar{B}}})}+B_4^{*}e^{-\beta _{4}(x-\delta ^{{\bar{B}}})}+D_2^{0},&{} x>\delta ^{{\bar{B}}}, \end{array}\right. } \end{aligned}$$

where $B_3^{*}$ and $B_4^{*}$ are constants to be determined. After that, substituting (13) into (12) and using similar calculations as before, we get (4) and (5).

As usual, we assume that the entrepreneur has the option to default, and doing so, s/he maximizes the value $E_2(x)$ of equity. Following this way and using the smooth-pasting condition, we derive the optimal default threshold given by (6). $\square$

#### Remark 1

By intuition, we realize that if the firm never defaults, $D_2^0$ defined in (5) is the value of debt and $E_2^0$ defined in (3) is the value of equity. The first term at the right-hand side of the third equality in (3) accounts for the unlevered firm value if jumps never happen, the second term represents a deduction for serving debt, and the last term $(1-\rho _e)(1+\theta )\frac{\mu +\lambda \xi }{r^2}$ records the value adjusted for the possible jumps of the cash flow. The other items of (2) except $E_2^0$ reflect the correction in value resulting from the possible default of the firm.

### The Pricing after the First-Stage Investment but Prior to the Second-Stage Investment

During this period, the borrower harvests the earnings stream $(1-\rho _e)(x-C)$ if the project’s cash flow $x \in {\mathcal {D}}=(\delta ^{B},\delta ^{{\bar{I}}})$. Once the cash flow exits from this domain, the claimant gets nothing if *x* is less than default threshold $\delta ^B$ or get a ‘lump-sum payoff’ $(1-\psi )E_2(\delta _{\tau _{{\mathcal {D}}}})$, where $\tau _{{\mathcal {D}}}$ is the first time of the cash flow goes across second-stage investment threshold $\delta ^{{\bar{I}}}$.

As before, the value of equity and of debt are still the functions of the current cash flow *x*, which are denoted by $E_1(x)$ and $D_1(x)$, respectively.

We have the following proposition.

#### Proposition 3.2

For a given default threshold $\delta ^{B}$ (before second-stage investment) and second-stage investment threshold $\delta ^{{\bar{I}}}$, let $x \in {\mathcal {D}}=(\delta ^{B},\delta ^{{\bar{I}}})$ be the current cash flow and the first-stage investment has taken place but the second-stage investment not. Then the value $E_1(x)$ of equity is given by

14 $$\begin{aligned} E_1(x)=A_1e^{\beta _{1}(x-\delta ^{{\bar{I}}})}+A_2e^{\beta _{2}(x-\delta ^{{\bar{I}}})}+A_3e^{-\beta _{3}(x-\delta ^{B})}+A_4e^{-\beta _{4}(x-\delta ^{B})}+E_1^{0}, \end{aligned}$$

where $E_1^{0}\equiv \frac{(1-\rho _e)(x-C)}{r} +\frac{(1-\rho _e)(\mu +\lambda \xi )}{r^2}$, and constants $A_1\sim A_4$ are the solution of the system of linear equations

15 $$\begin{aligned} {\left\{ \begin{array}{ll} A_1e^{\beta _{1}(\delta ^{B}-\delta ^{{\bar{I}}})}+A_2e^{\beta _{2}(\delta ^{B}-\delta ^{{\bar{I}}})}+A_3+A_4+(1-\rho _e)\frac{\delta ^{B}-C}{r}+(1-\rho _e)\frac{\mu +\lambda \xi }{r^2}=0,\\ A_1+A_2+A_3e^{-\beta _{3}(\delta ^{{\bar{I}}}-\delta ^{B})}+A_4e^{-\beta _{4}(\delta ^{{\bar{I}}}-\delta ^{B})}+(1-\rho _e)\left( \frac{\delta ^{{\bar{I}}}-C}{r}+\frac{\mu +\lambda \xi }{r^2}\right) =(1 - \psi )E_2(\delta ^{{\bar{I}}}) ,\\ \frac{A_1}{\beta _{1}+\eta _{2}}e^{\beta _{1}(\delta ^{B}-\delta ^{{\bar{I}}})}+\frac{A_2}{\beta _{2}+\eta _{2}}e^{\beta _{2}(\delta ^{B}-\delta ^{{\bar{I}}})}+\frac{A_3}{\eta _{2}-\beta _{3}}+\frac{A_4}{\eta _{2}-\beta _{4}}+\frac{1-\rho _e}{\eta _{2}}\left( \frac{\delta ^{B}-C}{r}-\frac{1}{r\eta _{2}}+\frac{\mu +\lambda \xi }{r^2}\right) =0,\\ \frac{A_1}{\beta _{1}-\eta _{1}}+\frac{A_2}{\beta _{2}-\eta _{1}}-\frac{A_3}{\beta _{3}+\eta _{1}}e^{-\beta _{3}(\delta ^{{\bar{I}}}-\delta ^{B})}-\frac{A_4}{\beta _{4}+\eta _{1}}e^{-\beta _{4}(\delta ^{{\bar{I}}}-\delta ^{B})}=\frac{1-\rho _e}{\eta _{1}}\left[ \frac{\delta ^I}{r}+\frac{1}{r\eta _{1}}-\frac{C}{r}+\frac{\mu +\lambda \xi }{r^2}\right] \\ -(1-\psi )\left[ \frac{A_3^*}{\beta _{3}+\eta _{1}}e^{-\beta _{3}(\delta ^{{\bar{I}}}-\delta ^{{\bar{B}}})}+\frac{A_4^*}{\beta _{4}+\eta _{1}}e^{-\beta _{4}(\delta ^{{\bar{I}}}-\delta ^{{\bar{B}}})}+\frac{(1-\rho _e)(1+\theta )}{\eta _{1}}\left( \frac{\delta ^{{\bar{I}}}}{r}+ \frac{1}{r\eta _{1}}-\frac{C}{r(1+\theta )}+\frac{\mu +\lambda \xi }{r^2}\right) \right] .\\ \end{array}\right. } \end{aligned}$$

The value $D_1(x)$ of debt is given by

16 $$\begin{aligned} D_1(x)=B_1e^{\beta _{1}(x-\delta ^{{\bar{I}}})}+B_2e^{\beta _{2}(x-\delta ^{{\bar{I}}})}+B_3e^{-\beta _{3}(x-\delta ^{B})}+B_4e^{-\beta _{4}(x-\delta ^{B})}+D_1^{0}, \end{aligned}$$

where $D_1^{0}\equiv \frac{(1-\rho _i)C}{r}$ and constants $B_1\sim B_4$ are the solution of the system of linear equations

17 $$\begin{aligned} {\left\{ \begin{array}{ll} \frac{B_1}{\beta _{1}-\eta _{1}}+\frac{B_2}{\beta _{2}-\eta _{1}}-\frac{B_3}{\beta _{3}+\eta _{1}}e^{-\beta _{3}(\delta ^{{\bar{I}}}-\delta ^{B})}-\frac{B_4}{\beta _{4}+\eta _{1}}e^{-\beta _{4}(\delta ^{{\bar{I}}}-\delta ^{B})}\\ =- \frac{B^{*}_3}{\beta _{3}+\eta _{1}}e^{-\beta _{3}(\delta ^{{\bar{I}}}-\delta ^{{\bar{B}}})}-\frac{B^{*}_4}{\beta _{4}+\eta _{1}}e^{-\beta _{4}(\delta ^{{\bar{I}}}-\delta ^{{\bar{B}}})}, \\ \frac{B_1\eta _{2}}{\beta _{1}+\eta _{2}}e^{\beta _{1}(\delta ^{B}-\delta ^{{\bar{I}}})}+\frac{B_2\eta _{2}}{\beta _{2}+\eta _{2}}e^{\beta _{2}(\delta ^{B}-\delta ^{{\bar{I}}})}+\frac{B_3\eta _{2}}{\eta _{2}-\beta _{3}}+\frac{B_4\eta _{2}}{\eta _{2}-\beta _{4}}\\ =\left[ \frac{(1-\rho _e)(1-\alpha )(\mu +\lambda \xi +r\delta ^{B})}{r^2}-\frac{(1-\rho _e)(1-\alpha )}{r\eta _{2}}(1-e^{-\eta _{2}(\delta ^{B}-\delta ^{{\bar{B}}}_0)} ) \right] -\frac{(1-\rho _i)C}{r},\\ B_1e^{\beta _{1}(\delta ^{B}-\delta ^{{\bar{I}}})}+B_2e^{\beta _{2}(\delta ^{B}-\delta ^{{\bar{I}}})}+B_3+B_4+\frac{(1-\rho _i)C}{r}=(1-\rho _e)(1-\alpha )\frac{\mu +\lambda \xi +r\delta ^{B}}{r^2},\\ B_1+B_2+B_3e^{-\beta _{3}(\delta ^{{\bar{I}}}-\delta ^{B})}+B_4e^{-\beta _{4}(\delta ^{{\bar{I}}}-\delta ^{B})}=B^{*}_3e^{-\beta _{3}(\delta ^{{\bar{I}}}-\delta ^{{\bar{B}}})}+B^{*}_4e^{-\beta _{4}(\delta ^{{\bar{I}}}-\delta ^{{\bar{B}}})}. \end{array}\right. } \end{aligned}$$

#### Proof

According to the aforementioned cash flow generated by equity, the equity value is

$$\begin{aligned} E_1(x) = {\mathbb {E}} \left[ \int _{0}^{\tau _{{\mathcal {D}}}} e^{-rs}(1-\rho _e)(\delta _s - C)\, ds + e^{-r \tau _{{\mathcal {D}}}}G(\delta _{\tau _{{\mathcal {D}}}}) \,\mid \,\delta _0=x \right] \end{aligned}$$

for $x\in {\mathcal {D}}=(\delta ^{B},\delta ^{{\bar{I}}})$, where function $G(\cdot )$ is given by

$$\begin{aligned} G(x)= {\left\{ \begin{array}{ll} 0, &{}\quad x\le \delta ^{B},\\ (1 - \psi )E_2(x),&{}\quad x\ge \delta ^{{\bar{I}}}.\\ \end{array}\right. } \end{aligned}$$

According to the proof method for Prop. 3.1, fuction $E_1(x)$ satisfies

18 $$\begin{aligned}&\mu \frac{\partial E_1(x)}{\partial x}+\frac{\sigma ^2}{2}\times \frac{\partial ^2 E_1(x)}{\partial x^2}+(1-\rho _e)(x - C)-(\lambda +r)E_1(x)\nonumber \\&\quad =-\lambda \left[ \int _{\delta ^{B}-x}^{\delta ^{{\bar{I}}}-x} E_1(x+z) h(z)dz+\int _{\delta ^{{\bar{I}}}-x}^{+\infty } (1 - \psi ) E_2(x+z) h(z)dz\right]. \end{aligned}$$

As before, we guess that $E_1(x)$ has the form of (14), where constants $A_1\sim A_4$ are to be determined. Then, we have

19 $$\begin{aligned}&\int _{0}^{\delta ^{{\bar{I}}}-x}E_1(x+z) p\cdot \eta _{1}e^{-\eta _{1}z}dz\nonumber \\&\quad = \frac{p\eta _{1}A_1}{\beta _{1}-\eta _{1}}\left( e^{\eta _{1}(x-\delta ^{{\bar{I}}})}-e^{\beta _{1}(x-\delta ^{{\bar{I}}})}\right) +\frac{p\eta _{1}A_2}{\beta _{2}-\eta _{1}}\left( e^{\eta _{1}(x-\delta ^{{\bar{I}}})}-e^{\beta _{2}(x-\delta ^{{\bar{I}}})}\right) \nonumber \\&\qquad -\frac{p\eta _{1}A_3}{\beta _{3}+\eta _{1}}\left( e^{\beta _{3}(\delta ^{B}-\delta ^{{\bar{I}}})}e^{\eta _{1}(x-\delta ^{{\bar{I}}})}-e^{-\beta _{3}(x-\delta ^{B})}\right) \nonumber \\&\qquad -\frac{p\eta _{1}A_4}{\beta _{4}+\eta _{1}}\left( e^{\beta _{4}(\delta ^{B}-\delta ^{{\bar{I}}})}e^{\eta _{1}(x-\delta ^{{\bar{I}}})}-e^{-\beta _{4}(x-\delta ^{B})}\right) \nonumber \\&\qquad +\frac{(1-\rho _e)p}{r}\left[ \left( C-\frac{\mu +\lambda \xi }{r}-\delta ^{{\bar{I}}}-\frac{1}{\eta _1}\right) e^{\eta _{1}(x-\delta ^{{\bar{I}}})} +x-C-\frac{\mu +\lambda \xi }{r}+\frac{1}{\eta _{1}}\right] \end{aligned}$$

and

20 $$\begin{aligned}&\int ^{0}_{\delta ^{B}-x}E_1(x+z) q\cdot \eta _{2}e^{\eta _{2}z}dz\nonumber \\&\quad =\frac{q\eta _{2}A_1}{\beta _{1}+\eta _{2}}\left( e^{\beta _{1}(x-\delta ^{{\bar{I}}})}-e^{\beta _{1} (\delta ^{B}-\delta ^{{\bar{I}}})}e^{-\eta _{2}(x-\delta ^{B})}\right) + \frac{q\eta _{2}A_2}{\beta _{2}+\eta _{2}}\nonumber \\&\qquad \left( e^{\beta _{2}(x-\delta ^{{\bar{I}}})}-e^{\beta _{2}(\delta ^{B}-\delta ^{{\bar{I}}})}e^{-\eta _{2}(x-\delta ^{B})}\right) \nonumber \\&\qquad - \frac{q\eta _{2}A_3}{\beta _{3}-\eta _{2}}\left( e^{-\beta _{3}(x-\delta ^{B})}-e^{-\eta _{2}(x-\delta ^{B})}\right) -\frac{q\eta _{2}A_4}{\beta _{4}-\eta _{2}}\left( e^{-\beta _{4}(x-\delta ^{B})}-e^{-\eta _{2}(x-\delta ^{B})}\right) \nonumber \\&\qquad +\frac{(1-\rho _e)q}{r}\left[ \left( C-\frac{\mu +\lambda \xi }{r}-\delta ^{B}+\frac{1}{\eta _2}\right) e^{-\eta _{2}(x-\delta ^{B})} +x-C-\frac{\mu +\lambda \xi }{r}-\frac{1}{\eta _{2}}\right] . \end{aligned}$$

In addition, we conclude from (2) that

21 $$\begin{aligned} \begin{array}{l} \int _{\delta ^{{\bar{I}}}-x}^{+\infty } (1 - \psi )E_2(x+z) p\cdot \eta _{1}e^{-\eta _{1}z}dz\\ =(1 - \psi )\left[ \frac{p\eta _{1}A_3^*}{\eta _{1}+\beta _{3}}e^{\eta _{1}(x-\delta ^{{\bar{I}}})+\beta _{3}(\delta ^{B}-\delta ^{{\bar{I}}})}+\frac{p\eta _{1}A_4^*}{\eta _{1}+\beta _{4}}e^{\eta _{1}(x-\delta ^{{\bar{I}}})+\beta _{4}(\delta ^{B}-\delta ^{{\bar{I}}})}\right] \\ \ \ \ +\frac{(1 - \psi )(1-\rho _e)(1+\theta )p}{r} \left[ \delta ^{{\bar{I}}}+\frac{1}{\eta _{1}}-\frac{C}{1+\theta }+\frac{\mu +\lambda \xi }{r}\right] e^{\eta _{1}(x-\delta ^{{\bar{I}}})}. \end{array} \end{aligned}$$

Similar to the proof of Prop. 3.1 and using a value-matching condition, we get (15) by substituting (19), (20) and (21) into (18).

In the same way, the value $D_1(x)$ of debt is given by

$$\begin{aligned} D_1(x)={\mathbb {E}} \left[ \int _{0}^{\tau _{{\mathcal {D}}}} e^{-rs} (1-\rho _i)C \,ds +e^{-r\tau _{{\mathcal {D}}}}G(\delta _{\tau _{{\mathcal {D}}}})\, \mid \,\delta _0=x\right] , \quad x\in {\mathcal {D}}=(\delta ^{B},\delta ^{{\bar{I}}}), \end{aligned}$$

where function $G(\cdot )$ is defined by

$$\begin{aligned} G(x)\equiv {\left\{ \begin{array}{ll} \left[ (1-\rho _e)(1-\alpha )\frac{\mu +\lambda \xi +rx}{r^2}\right] \vee 0,&{}\quad x\le \delta ^{B}, \\ D_2(x),&{}\quad x\ge \delta ^{{\bar{I}}}. \end{array}\right. } \end{aligned}$$

Noting the previous assumptions and following Yang and Zhang (2015a), we guess that

$$\begin{aligned} D_1(x)={\left\{ \begin{array}{ll} 0; &{} x \le \delta ^{{\bar{B}}}_0.\\ (1-\rho _e)(1-\alpha )\frac{\mu +\lambda \xi +rx}{r^2}; &{} \delta ^{{\bar{B}}}_0< x\le \delta ^{B}.\\ B_1e^{\beta _{1}(x-\delta ^{{\bar{I}}})}+B_2e^{\beta _{2}(x-\delta ^{{\bar{I}}})}+B_3e^{-\beta _{3}(x-\delta ^{B})}+B_4e^{-\beta _{4}(x-\delta ^{B})}+D_1^{0}; &{} \delta ^{B}< x< \delta ^{{\bar{I}}}.\\ D_2(x); &{} x\ge \delta ^{{\bar{I}}}. \end{array}\right. } \end{aligned}$$

Thanks to Bellman’s principle of optimization, we get that debt value $D_1(x)$ satisfies the following equation:

$$\begin{aligned}&\mu \frac{\partial D_1(x)}{\partial x}+\frac{\sigma ^{2}\partial ^2 D_1(x)}{2\partial x^2}+(1-\rho _i)C\nonumber \\&\qquad +\lambda \int _{\delta ^{{\bar{B}}}_0-x}^{\delta ^{B}-x} (1-\rho _e)(1-\alpha )\frac{\mu +\lambda \xi +r(x+z)}{r^2} h(z)dz\nonumber \\&\quad =(r+\lambda )D_1(x)-\lambda \int _{\delta ^{B}-x}^{\delta ^{{\bar{I}}}-x} D_1(x+z) h(z)dz-\lambda \int _{\delta ^{{\bar{I}}}-x}^{+\infty } D_2(x+z) h(z)dz. \end{aligned}$$

And similarly, we get (17) by comparing the coefficients of exponential terms. $\square$

From (14), we conclude that the value of equity is determined by the default threshold $\delta ^{B}$ and second-stage investment threshold $\delta ^{{\bar{I}}}$ in addition to the cash flow level when the first-stage investment is exercised. The default threshold is naturally decided by the borrower, whose aim is to maximize the value of equity. The send-stage investment threshold can be decided by the entrepreneur or the insurer. Since the computation methods for them are similar to each other, to save space, we assume the growth (second-stage) investment threshold is decided by the borrower in the following text.

At the end of this section, we use a smooth-pasting condition to derive the following optimal second-stage investment threshold and optimal default threshold if the second-stage investment is not conducted yet. The conclusions are summarized in the following theorem.

#### Theorem 3.3

If the first-stage investment is exercised but the second-stage investment is not, the borrower’s optimal bankruptcy threshold $\delta ^{B}$ and optimal second-stage investment threshold $\delta ^{{\bar{I}}}$ are a solution of the following system of equations:

22 $$\begin{aligned} {\left\{ \begin{array}{ll} A_1\beta _{1}e^{\beta _{1}(\delta ^{B}-\delta ^{{\bar{I}}})}+A_2e^{\beta _{2}(\delta ^{B}-\delta ^{{\bar{I}}})}-A_3\beta _{3}-A_4\beta _{4}+\frac{(1-\rho _e)}{r}=0,\\ A_1\beta _{1}+A_2\beta _{2}-A_3\beta _{3}e^{-\beta _{3}(\delta ^{{\bar{I}}}-\delta ^{B})}-A_4\beta _{4}e^{-\beta _{4}(\delta ^{{\bar{I}}}-\delta ^{B})}+\frac{(1-\rho _e)}{r}\\ =(1-\psi )\left[ -A_3^{*}\beta _{3}e^{-\beta _{3}(\delta ^{{\bar{I}}}-\delta ^{{\bar{B}}})}-A_4^{*}\beta _{4}e^{-\beta _{4}(\delta ^{{\bar{I}}}-\delta ^{{\bar{B}}})}+(1+\theta )\frac{(1-\rho _e)}{r}\right] , \end{array}\right. } \end{aligned}$$

where constants $A_3^{*}$ and $A_4^{*}$ are given in Proposition 3.1 and variables $A_1\sim A_4$ are a function of thresholds $\delta ^{B}$ and $\delta ^{{\bar{I}}}$ given by (15).

#### Proof

The lemma is easily concluded from the following smooth-pasting conditions:

$$\begin{aligned} \frac{\partial E_1(x)}{\partial x}\mid _{{\delta ^B}+}=0,\qquad \frac{\partial E_1(x)}{\partial x}\mid _{\delta ^{{\bar{I}}}-}=(1-\psi )\frac{\partial E_2(x)}{\partial x}\mid _{\delta ^{{\bar{I}}}+}, \end{aligned}$$

where the left-hand sides of the two equations represent the right derivative at $\delta ^B$ and the left derivative at $\delta ^{{\bar{I}}}$ of the function $E_1(\cdot )$ respectively. Such notations are used at the right-hand side of the second equality and throughout the text. $\square$

## The Pricing and Timing of the Option to make the First-Stage Investment with the G-I Mode

We now turn to the first-stage investment. For this aim, we must fix the value of the forward-like claim and define the fair guarantee at the same time. Doing so, we assume that the guarantee market is so competitive that the net present value of insurers is zero.

*The forward-like claim’s value* According to the G-I mode recently invented by HTI, the second-stage investment cost ${\bar{I}}$ is financed by insurers. In return, borrowers agree to grant fraction $\psi$ of equity to insurers. As a result, at the first-stage investment time, insurers obtain the forward-like claim with its value being $\psi E_2(\delta _{\tau _{{\mathcal {D}}}})- {\bar{I}}$ at the second-stage investment time $\tau _{{\mathcal {D}}}$. Here ${\mathcal {D}}\equiv \left( \delta ^{B}\,,\, \delta ^{{\bar{I}}}\right)$ and $\tau _{{\mathcal {D}}}$ is the first departure time of the project’s cash flow from domain ${\mathcal {D}}$ as defined by Dong and Yang (2020).

Following the asset pricing theory, we conclude the following proposition.

### Proposition 4.1

Let’s denotes the current cash flow level is *x*. For a given second-stage investment threshold $\delta ^{{\bar{I}}}$, a given default threshold $\delta ^B$, $\delta ^I>\delta ^B$, and a certain $\psi$. The value *FL*(*x*) of the forward-like claim defined by the fraction $\psi$ of equity and the second-stage investment cost ${\bar{I}}$ is given by

23 $$\begin{aligned} FL(x)= {\left\{ \begin{array}{ll} L_1e^{\beta _{1}(x-\delta ^{{\bar{I}}})}+L_2e^{\beta _{2}(x-\delta ^{{\bar{I}}})}+L_3e^{-\beta _{3}(x-\delta ^{B})}+L_4e^{-\beta _{4}(x-\delta ^{B})}, &{} x\in {\mathcal {D}}\\ \psi E_2(x)- {\bar{I}},&{} x\ge \delta ^{{\bar{I}}},\\ 0,&{} x\le \delta ^{B}, \end{array}\right. } \end{aligned}$$

where constants $L_1\sim L_4$ are the solution of the following system of linear equations:

24 $$\begin{aligned} {\left\{ \begin{array}{ll} L_1e^{\beta _{1}(\delta ^{B}-\delta ^{{\bar{I}}})}+L_2e^{\beta _{2}(\delta ^{B}-\delta ^{{\bar{I}}})}+L_3+L_4=0,\\ L_1+L_2+L_3e^{-\beta _{3}(\delta ^{{\bar{I}}}-\delta ^{B})}+L_4e^{-\beta _{4}(\delta ^{{\bar{I}}}-\delta ^{B})}=\psi E_2(\delta ^{{\bar{I}}})-{\bar{I}} ,\\ \frac{L_1}{\beta _{1}+\eta _{2}}e^{\beta _{1}(\delta ^{B}-\delta ^{{\bar{I}}})}+\frac{L_2}{\beta _{2}+\eta _{2}}e^{\beta _{2}(\delta ^{B}-\delta ^{{\bar{I}}})}+\frac{L_3}{\eta _{2}-\beta _{3}}+\frac{L_4}{\eta _{2}-\beta _{4}} =0,\\ \frac{L_1}{\beta _{1}-\eta _{1}}+\frac{L_2}{\beta _{2}-\eta _{1}}-\frac{L_3}{\beta _{3}+\eta _{1}}e^{-\beta _{3}(\delta ^{{\bar{I}}}-\delta ^{B})}-\frac{L_4}{\beta _{4}+\eta _{1}}e^{-\beta _{4}(\delta ^{{\bar{I}}}-\delta ^{B})}-\frac{{\bar{I}}}{\eta _{1}}\\ = -\psi \left[ \frac{P_1}{\beta _{3}+\eta _{1}}e^{-\beta _{3}(\delta ^{{\bar{I}}}-\delta ^{{\bar{B}}})}+\frac{P_2}{\beta _{4}+\eta _{1}}e^{-\beta _{4}(\delta ^{{\bar{I}}}-\delta ^{{\bar{B}}})}+\frac{(1-\rho _e)(1+\theta )}{\eta _{1}}\left( \frac{\delta ^{{\bar{I}}}}{r}+ \frac{1}{r\eta _{1}}-\frac{C}{r(1+\theta )}+\frac{\mu +\lambda \xi }{r^2}\right) \right] . \end{array}\right. } \end{aligned}$$

### Proof

Clearly, it suffices to prove the first equality of (23). Define function

$$\begin{aligned} G(x)\equiv {\left\{ \begin{array}{ll} \psi E_2(x)- {\bar{I}},&{}\hbox {if }x\ge \delta ^{{\bar{I}}},\\ 0,&{}\hbox {if }x\le \delta ^{B}, \end{array}\right. } \end{aligned}$$

and then the value of the forward-like claim is

$$\begin{aligned} FL(x)={\mathbb {E}}\left[ e^{-r\tau _{{\mathcal {D}}}}G(\delta _{\tau _{{\mathcal {D}}}})\right] ,\ x\in (\delta ^B, \delta ^{{\bar{I}}}). \end{aligned}$$

Using a method similar to Bellman’s principle of optimization, we derive that *FL*(*x*) satisfies the following equation:

25 $$\begin{aligned}&\mu \frac{\partial FL(x)}{\partial x}+\frac{\sigma ^{2}\partial ^2 FL(x)}{2\partial x^2}-(r+\lambda )FL(x)\nonumber \\&\quad =-\lambda \left[ \int _{\delta ^{B}-x}^{\delta ^{{\bar{I}}}-x} FL(x+z) h(z)dz+\int _{\delta ^{{\bar{I}}}-x}^{+\infty } \left( \psi E_2(x+z)-{\bar{I}}\right) h(z)dz\right] . \end{aligned}$$

Similar to the proof of Prop 3.2. We guess that value *FL*(*x*) has the form of the first equality in (23) and then substitute it into (25). After a tedious computation, we get (24) through comparting the coefficients of the exponential terms and using a value-matching condition. $\square$

As argued by Dong and Yang (2020), many projects, particularly high-tech ones, are promising and the forward-like claim would be therefore considerably valuable, though the values of some projects are possibly negative. In short, the financing mode with the trial-investment stages is a great innovation since it significantly decreases the investment risk both for entrepreneurs and insurers. What’s more, it effectively deals with the provision regulated by Chinese government that the guarantee fee rate should not be higher than 3% or so.

*The fair guarantee.* To support MSMEs, Chinese governments demand that the guarantee fee rate be less than 3% or so but this requirement would make insurers reluctant to enter into the agreement due to MSMEs’ high default risk. To solve this problem, HTI invents the G-I mode defined by pair $(g, \psi )$.

According to the pecking order theory, an entrepreneur should borrow as little as possible. We therefore assume that the amount of money borrowed equals the sunk cost *I* plus the guarantee fee as did in Dong and Yang (2020), i.e. the loan defined by the coupon rate *C* satisfies

26 $$\begin{aligned} (1-g)\frac{(1-\rho _i)C}{r}=I, \end{aligned}$$

where $(1-\rho _i)C/r$ is actually the amount of the money borrowed.

Next, we determine the fraction $\psi$ which is actually specified by solving a Nash equilibrium. In sharp contrast to prior papers on credit guarantees, similar to a loan commitment addressed by Mauer and Sarkar (2005) and Song and Yang (2016), the guarantee costs defined by pair $(g, \psi )$ are contracted before investment. Intuitively and as implied by previous derivations, all the values of firm securities, investment thresholds, and default threshold are totally determined by pair $(g, \psi )$. On the other hand, for a given guarantee fee rate *g*, the fraction $\psi$ is fully determined by the two investment thresholds and two default thresholds. Going one step further, we conclude that for a given fee rate *g*, the fair fraction $\psi$ and all the optimal threshold constitute a Nash equilibrium of the game between the borrower and insurer.

Specifically, if all the investment thresholds and default thresholds are given, to make the guarantee fair, the fraction $\psi$ of equity granted to insurers must satisfy the following equality:

27 $$\begin{aligned} {\mathbb {E}}\left[ \exp (-r\tau _I)\left( D_1(\delta _{\tau _I})+FL(\delta _{\tau _I})-(1-g)\frac{(1-\rho _i)C}{r}\right) \right] =0, \end{aligned}$$

where $\tau _{I}$ is the first time of the cash flow surpassing the first-stage investment threshold $\delta ^I$, i.e. the stopping time $\tau _{I}\equiv \inf \left\{ t\ge 0: \delta _{t}\ge \delta ^I\right\}$ for a given investment threshold $\delta ^I$. This equality means that the net present value of the guarantee agreement is zero and thus it is fair. Thanks to (26), the left-hand side of (27) can be considered as the value of a forward at time zero when the guarantee agreement is signed, of which the forward price is *I* and the underlying asset price is $D_1(\delta _{t})+FL(\delta _{t})$. The forward value is determined by the current cash flow level, i.e. $\delta _0=x$ and we denote it by $V_F(x)$. To derive the fair fraction $\psi$, we must fix its value. We have

### Proposition 4.2

Let $\delta _0=x$. The value of the forward defined by $D_1(\delta _{t})+FL(\delta _{t})-I$ with the exercising threshold being $\delta ^I$ is given by

28 $$\begin{aligned} V_F(x)= \left\{ \begin{array}{ll} D_1(x)+FL(x)-I, &{}\hbox {if }x\ge \delta ^I,\\ K_1e^{\beta _{1}(x-\delta ^I)}+K_2e^{\beta _{2}(x-\delta ^I)}, &{}\hbox {if }x<\delta ^I, \end{array} \right. \end{aligned}$$

where constants $K_1$ and $K_2$ constitute the solution of the following system of linear equations

29 $$\begin{aligned} {\left\{ \begin{array}{ll} K_1+K_2=D_1(\delta ^I)+FL(\delta ^I)-I,\\ \frac{K_1-\varGamma _1e^{\beta _{1}(\delta ^I-\delta ^{{\bar{I}}})}}{\beta _{1}-\eta _{1}}+\frac{K_2-\varGamma _2e^{\beta _{2}(\delta ^I-\delta ^{{\bar{I}}})}}{\beta _{2}-\eta _{1}}+\frac{(1-\rho _i)C}{r\eta _{1}}= \frac{I}{\eta _{1}}-\frac{\varGamma _3e^{-\beta _{3}(\delta ^I-\delta ^{B})}}{\beta _{3}+\eta _{1}}-\frac{\varGamma _4e^{-\beta _{4}(\delta ^I-\delta ^{B})}}{\beta _{4}+\eta _{1}}, \end{array}\right. } \end{aligned}$$

and $\varGamma _i\equiv B_i+L_i$, $B_i$ is given by (17) and $L_i$ is given by (24) for $i=1,2,3,4$.

### Proof

According to the definition of the stopping time $\tau _{I}$ in (27), we have

$$\begin{aligned} V_F(x)= & {} {\mathbb {E}}\left[ e^{-r\tau _{I}}\left( D_1(\delta _{\tau _I})+FL(\delta _{\tau _{I}})-I\right) \right] . \end{aligned}$$

Thanks to the idea of Bellman’s principle of optimization, we derive

30 $$\begin{aligned}&\mu \frac{\partial V_F(x)}{\partial x}+\frac{\sigma ^{2}}{2}\times \frac{\partial ^2 V_F(x)}{\partial x^2}-(r+\lambda )V_F(x)+\lambda \int ^{\delta ^{I}-x}_{-\infty } V_F(x+z) h(z)dz\nonumber \\&\quad =-\lambda \left[ \int _{\delta ^I-x}^{+\infty } \left( D_1(x+z)+FL(x+z)-I\right) h(z)dz\right] . \end{aligned}$$

We guess that value $V_F(x)$ has the form of the second equality of (28), where $K_1$ and $K_2$ are constants to be determined. Substituting it into (30), we get (29) after tedious calculations and comparing the coefficients of the exponential terms. $\square$

*The pricing and timing of the option to make the first-stage investment.* Armed with previous discussions, we turn to the pricing and timing of the investment option in two stages. The conclusions are summarized in the following theorem.

### Theorem 4.3

For a given first-stage investment threshold $\delta ^I$, the value of the option to invest in the project with the growth opportunity is given by

31 $$\begin{aligned} F(x)= \left\{ \begin{array}{ll} E_1(x)+D_1(x)+FL(x)-I, &{}\hbox {if }x\ge \delta ^I,\\ M_1e^{\beta _{1}(x-\delta ^I)}+M_2e^{\beta _{2}(x-\delta ^I)}, &{}\hbox {if }x<\delta ^I, \end{array} \right. \end{aligned}$$

where *x* is the current cash flow level, constants $M_1$ and $M_2$ are the solution of the following system of linear equations

32 $$\begin{aligned} {\left\{ \begin{array}{ll} M_1+M_2=E_1(\delta ^I)+D_1(\delta ^I)+FL(\delta ^I)-I,\\ \frac{M_1}{\beta _{1}-\eta _{1}}+\frac{M_1}{\beta _{2}-\eta _{1}}=\frac{(\rho _i-\rho _e)C}{r\eta _{1}}-\frac{(1-\rho _e)}{\eta _{1}}\left[ \frac{\delta ^I}{r}+\frac{1}{r\eta _{1}}+\frac{\mu +\lambda \xi }{r^2}\right] +\frac{I}{\eta _{1}}\\ +\frac{\varGamma _1+A_1}{\beta _{1}-\eta _{1}}e^{\beta _{1}(\delta ^I-\delta ^{{\bar{I}}})}+\frac{\varGamma _2+A_2}{\beta _{2}-\eta _{1}}e^{\beta _{2}(\delta ^I-\delta ^{{\bar{I}}})}-\frac{\varGamma _3+A_3}{\beta _{3}+\eta _{1}}e^{-\beta _{3}(\delta ^I-\delta ^{B})}-\frac{\varGamma _4+A_4}{\beta _{4}+\eta _{1}}e^{-\beta _{4}(\delta ^I-\delta ^{B})} \end{array}\right. } \end{aligned}$$

while *I* is the sunk cost and $\varGamma _i$ is mentioned in (29), with $A_i$ given by (15), $i=1,2,3,4$.

### Proof

We note that the project value at the first-stage investment time is $E_1(\delta _{\tau _{I}})+D_1(\delta _{\tau _I})+FL(\delta _{\tau _{I}})-I$, where $\delta _{\tau _I}$ is the cash flow level at the investment time $\tau _I$. Consequently, the value of the option to invest in the project is given by

$$\begin{aligned} F(x)= & {} {\mathbb {E}}\left[ e^{-r\tau _{I}}\left( E_1(\delta _{\tau _{I}})+D_1(\delta _{\tau _I})+FL(\delta _{\tau _{I}})-I\right) \right] . \end{aligned}$$

Therefore, it is clear that the first equality of (31) holds true. To prove the second equality, according to Bellman’s principle of optimization, we derive that the value *F*(*x*) of the option satisfies

33 $$\begin{aligned}&\mu \frac{\partial F(x)}{\partial x}+\frac{\sigma ^{2}}{2}\times \frac{\partial ^2 F(x)}{\partial x^2}-(r+\lambda )F(x)+\lambda \int ^{\delta ^{I}-x}_{-\infty } F(x+z) h(z)dz\nonumber \\&\quad =-\lambda \left[ \int _{\delta ^I-x}^{+\infty } \left( E_1(x+z)+D_1(x+z)+FL(x+z)-I\right) h(z)dz\right] . \end{aligned}$$

We guess that value *F*(*x*) has the form of the second equality of (31), where $M_1$ and $M_2$ are constants to be determined. Substituting it into (33), we get (32) after tedious calculations and comparing the coefficients of the exponential terms as did before. $\square$

We now use the smooth-pasting condition to fix the optimal investment threshold. The conclusion is shown below.

### Theorem 4.4

Suppose the cash flow level $\delta$ is given by (1) and it increases to $(1+\theta )\delta$ once the second-stage investment is executed. The sunk cost is *I*, which is totally borrowed from a bank under a guarantee provided by an insurer. The guarantee is defined by pair $(g, \psi )$ as determined at the time of the agreement, where ${\bar{I}}$ is the second-stage investment cost totally paid by the insurer. Then the optimal first-stage investment threshold $\delta ^I$ is a solution of the following nonlinear equation:

34 $$\begin{aligned} \begin{array}{l} M_1+M_2-\frac{(1-\rho _e)}{r}\\ =\varGamma _1\beta _{1} e^{\beta _{1}(\delta ^I-\delta ^{{\bar{I}}})}+\varGamma _2\beta _{2} e^{\beta _{2}(\delta ^I-\delta ^{{\bar{I}}})}-\varGamma _3\beta _{3} e^{-\beta _{3}(\delta ^I-\delta ^{B})}-\varGamma _4\beta _{4} e^{-\beta _{4}(\delta ^I-\delta ^{B})}, \end{array} \end{aligned}$$

where constants $M_i, i=1,2$ and $\Gamma _i, i=1,2,3,4$ are given by Theorem 4.3.

### Proof

The conclusion is derived from the following smooth-pasting condition:

$$\begin{aligned} \frac{\partial F(x)}{\partial x}\mid_{{\delta ^I}-}=\frac{\partial (E_1(x)+D_1(x)+FL(x))}{\partial x}\mid _{{\delta ^I}+}. \end{aligned}$$

$$\square $$

## The Algorithm and Examples for the Two-Stage Investment

As a summary of our work, we provide the following algorithm for the pricing and timing of the option to make the two-stage investment with credit guarantees. 
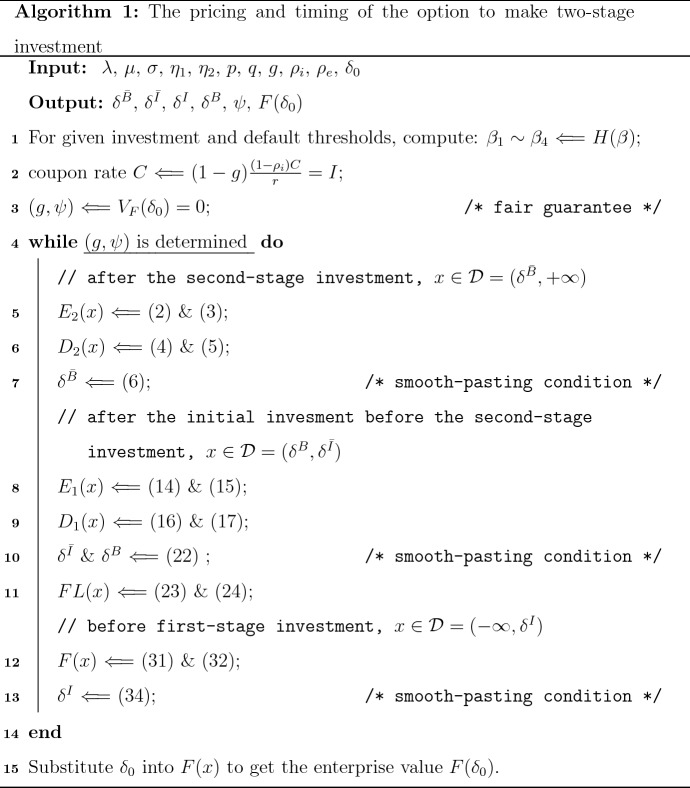

**Table 1:** The pricing and timing of the option $r=0.05$, $\rho _i=0.05$, $\rho _f=0.4$, $\mu =0.02$, $\sigma =0.35$, $\lambda =8$ $\eta _{1}=10$, $\eta _{2}=9$, $q=0.6$, $p=0.4$, $\alpha =0.35$, $I=20$, ${\bar{I}}=90$, $\delta _0=1$

$\theta$	*g*	$\psi$ (%)	$F(\delta _0)$	$\delta ^{I}$	$\delta ^{{\bar{I}}}$
0.45	$-$0.005	18.00	11.020124	10.140507	43.006994
	0.000	15.57	11.070912	10.137452	37.788696
	0.005	14.28	11.105132	10.134367	35.618675
	0.010	13.42	11.130458	10.131251	34.366575
	0.015	12.75	11.151866	10.128104	33.460672
	0.020	12.18	11.170919	10.124924	32.742706
	0.025	11.68	11.188355	10.121712	32.144130
	0.030	11.22	11.204595	10.118467	31.628584
	0.035	10.80	11.219904	10.115188	31.174371
	0.040	10.41	11.234465	10.111875	30.767378
	0.045	10.04	11.248408	10.108527	30.397934
0.5	$-$0.005	18.24	11.088645	10.140506	37.394661
	0.000	16.60	11.142845	10.137451	34.672155
	0.005	15.70	11.176507	10.134366	33.403728
	0.010	15.02	11.203766	10.131250	32.523241
	0.015	14.45	11.227515	10.128102	31.836887
	0.020	13.95	11.248967	10.124923	31.269315
	0.025	13.51	11.268765	10.121710	30.782702
	0.030	13.10	11.287304	10.118465	30.355041
	0.035	12.72	11.304844	10.115186	29.972401
	0.040	12.36	11.321569	10.111873	29.625340
	0.045	12.02	11.337613	10.108526	29.307151
0.55	$-$0.005	18.37	11.205477	10.140504	33.229049
	0.000	17.53	11.245464	10.137449	32.127821
	0.005	16.89	11.277504	10.134364	31.355244
	0.010	16.35	11.305254	10.131248	30.747977
	0.015	15.88	11.330214	10.128101	30.242537
	0.020	15.46	11.353179	10.124921	29.806817
	0.025	15.07	11.374629	10.121709	29.422158
	0.030	14.70	11.394882	10.118463	29.076659
	0.035	14.36	11.414159	10.115184	28.762226
	0.040	14.04	11.432623	10.111871	28.473089
	0.045	13.73	11.450399	10.108523	28.204974

Table 1 presents examples. It says that the value $F(\delta _0)$ of the option (i.e. the firm value) increases and the two investment thresholds decrease with the growth factor $\theta$. The fraction $\psi$ of equity decreases with the guarantee fee rate *g*. These numerical results are quite in agreement with intuition, and show that our algorithm is feasible and effective.

## Conclusion

The guarantee-investment financing mode (G-I mode) is recently invented by Chinese entrepreneurs and it has acquired great successes in overcoming the financing difficulty experienced by micro-, small- and medium-sized enterprises (MSMEs). However, there are no theories in the literature on the G-I financing mode in addition to Dong and Yang (2020).

In this paper, we develop a jump-diffusion model for the G-I mode instead of a pure diffusion one discussed by Dong and Yang (2020). The fraction of equity and guarantee fee rate are contracted before investment like a loan commitment considered by Mauer and Sarkar (2005) and Song and Yang (2016). We aim to produce an algorithm for the pricing and timing of the option to invest in a project with the G-I mode. We make use of the well-known theory of corporate securities pricing and capital structure, see e.g. Leland (1994), and the backward induction method, see e.g. Tan and Yang (2017), and provide closed-form solutions and produce a numerical algorithm for the timing and pricing of the option to make the two-stage investment.

We emphasize that in our model, the second-stage investment is initiated by borrowers (entrepreneurs). In practice, the second investment option might be owned by borrowers or insurers. To whom this option should be granted is an interesting problem, which is related to the optimal security design theory. We do not consider the problem here. Instead, we recommend seeing Dong and Yang (2020) for a discussion.

## Data Availability

It will be provided upon request.

## Footnotes

1 By the way, we point that Yang and Zhang (2015a) did not take into account the possible nagative value of the “surplus value” $\left[ (1-\rho _e)(1-\alpha )(1+\theta )\frac{\mu +\lambda \xi +rx}{r^2}\right]$ and therefore their conclusions should be accordingly corrected.

## Code Availability

It will be provided upon request.

## References

[CR1] Bachas N, Kim OS, Yannelis C (2021). Loan guarantees and credit supply. Journal of Financial Economics.

[CR2] Dixit AK, Dixit RK, Pindyck RS (1994). Investment under Uncertainty.

[CR3] Dong, L., & Yang, Z. (2020). Guarantee-investment combination financing contract design for entrepreneurship. Available at SSRN 3743153.

[CR4] Gan L, Luo P, Yang Z (2016). Real option, debt maturity and equity default swaps under negotiation. Finance Research Letters.

[CR5] Kou SG (2002). A jump-diffusion model for option pricing. Management science.

[CR6] Kou SG, Wang H (2003). First passage times of a jump diffusion process. Advances in applied probability.

[CR7] Leland HE (1994). Corporate debt value, bond covenants, and optimal capital structure. The journal of finance.

[CR8] Luo P, Wang H, Yang Z (2016). Investment and financing for smes with a partial guarantee and jump risk. European Journal of Operational Research.

[CR9] Luo P, Yang Z (2019). Growth option and debt maturity with equity default swaps in a regime-switching framework. Macroeconomic Dynamics.

[CR10] Mauer D, Sarkar S (2005). Real option, agency conflicts, and optimal capital structure. Journal of Banking & Finance.

[CR11] McDonald R, Siegel D (1986). The value of waiting to invest. The quarterly journal of economics.

[CR12] Merton RC (1976). Option pricing when underlying stock returns are discontinuous. Journal of financial economics.

[CR13] Merton RC (1977). An analytic derivation of the cost of deposit insurance and loan guarantees an application of modern option pricing theory. Journal of banking & finance.

[CR14] Myers SC (1977). Determinants of corporate borrowing. Journal of financial economics.

[CR15] Song D, Yang Z (2016). Contingent capital, real options and agency costs. International Review of Finance.

[CR16] Tan Y, Yang Z (2017). Growth option, contingent capital and agency conflicts. International Review of Economics & Finance.

[CR17] Tang X, Yang Z (2017). Optimal investment and financing with macroeconomic risk and loan guarantees. Journal of Credit Risk.

[CR18] Tang X, Yang Z (2018). Irreversible investment, ambiguity, and equity default swaps. Applied Economics Letters.

[CR19] Wang H, Yang Z, Zhang H (2015). Entrepreneurial finance with equity-for-guarantee swap and idiosyncratic risk. European Journal of Operational Research.

[CR20] Xiang H, Yang Z (2015). Investment timing and capital structure with loan guarantees. Finance Research Letters.

[CR21] Yang Z (2020). Investment and asset securitization with an option-for-guarantee swap. European Financial Management.

[CR22] Yang Z, Zhang C (2015). The pricing of two newly invented swaps in a jump-diffusion model. Annals of Economics and Finance.

[CR23] Yang Z, Zhang C (2015). Two new equity default swaps with idiosyncratic risk. International Review of Economics & Finance.

[CR24] Yang Z, Zhang H (2013). Optimal capital structure with an equity-for-guarantee swap. Economics Letters.

